# Supplementary material for: A native conjugative plasmid confers potential selective advantages to plant growth-promoting Bacillus velezensis strain GH1-13
Source: Commun Biol. 2021 May 14;4:582. doi: 10.1038/s42003-021-02107-z (PMC8121941; doi:10.1038/s42003-021-02107-z)
Supplement: Supplementary file 2 — Supplementary Information [file 42003_2021_2107_MOESM2_ESM.pdf]

Supplementary Information

Supplementary Figure 1. Multiple sequence alignment of *Bacillus velezensis* GH1-13 pBV71 plasmid (GenBank accession number CP019039), *B. safensis* U14-5 unnamed plasmid (CP015608), and four draft genome sequences of different *Bacillus* species: MBPE01000008 (*B. subtilis* MB378), NUFR01000009 (*B. cereus* AFS057690), NVGH01000017 (*B. wiedmannii* AFS079268), and NVPP01000024 (*B. pseudomycoides* AFS098564). In order to perform the genome alignment, contigs which have high similarity with pBV71 were downloaded from GenBank database. Genome synteny of the plasmids was compared using Mauve[1] software v 2.4.0. The light purple region (containing WP_077721662.1 to WP_077721667.1 in pBV71) was conserved at all contigs. The conserved region of 54–60kb in pBV71 encodes a Helicase HerA domain protein, NlpC/P60 family protein, and a putative conjugal transfer protein, Tra.

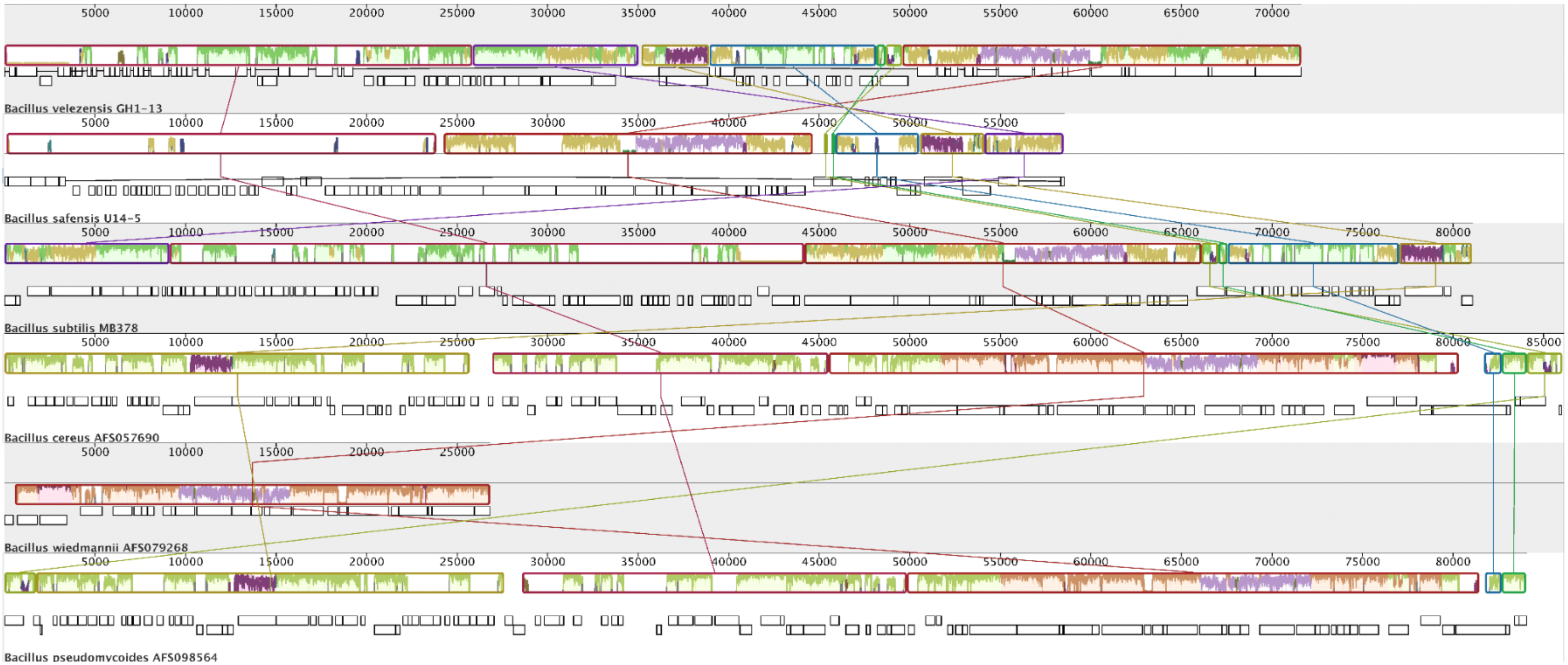
Bacillus velezensis GH1-13
Bacillus safensis U14-5
Bacillus subtilis MB378
Bacillus cereus AFS057690
Bacillus wiedmannii AFS079268
Bacillus pseudomycoides AFS098564

Supplementary Figure 2. Comparison of transcriptomes of pBV71 at different growth phases of host *B. velezensis* strain GH1-13. From outside to the center: genes on forward strand, CDS accession number, genes on reverse strand, and a heatmap with the expression values (TMM) of 5 h, 8 h, 12 h, and 24 h, respectively. The graphical circular map of the complete genome was constructed and visualized using Circos[2] version 0.67. BLAST searches of functionally predicted genes against NCBI[3], Uniprot[4], Pfam[5], and Subsystem[6] databases are shown in Supplementary Data 2.

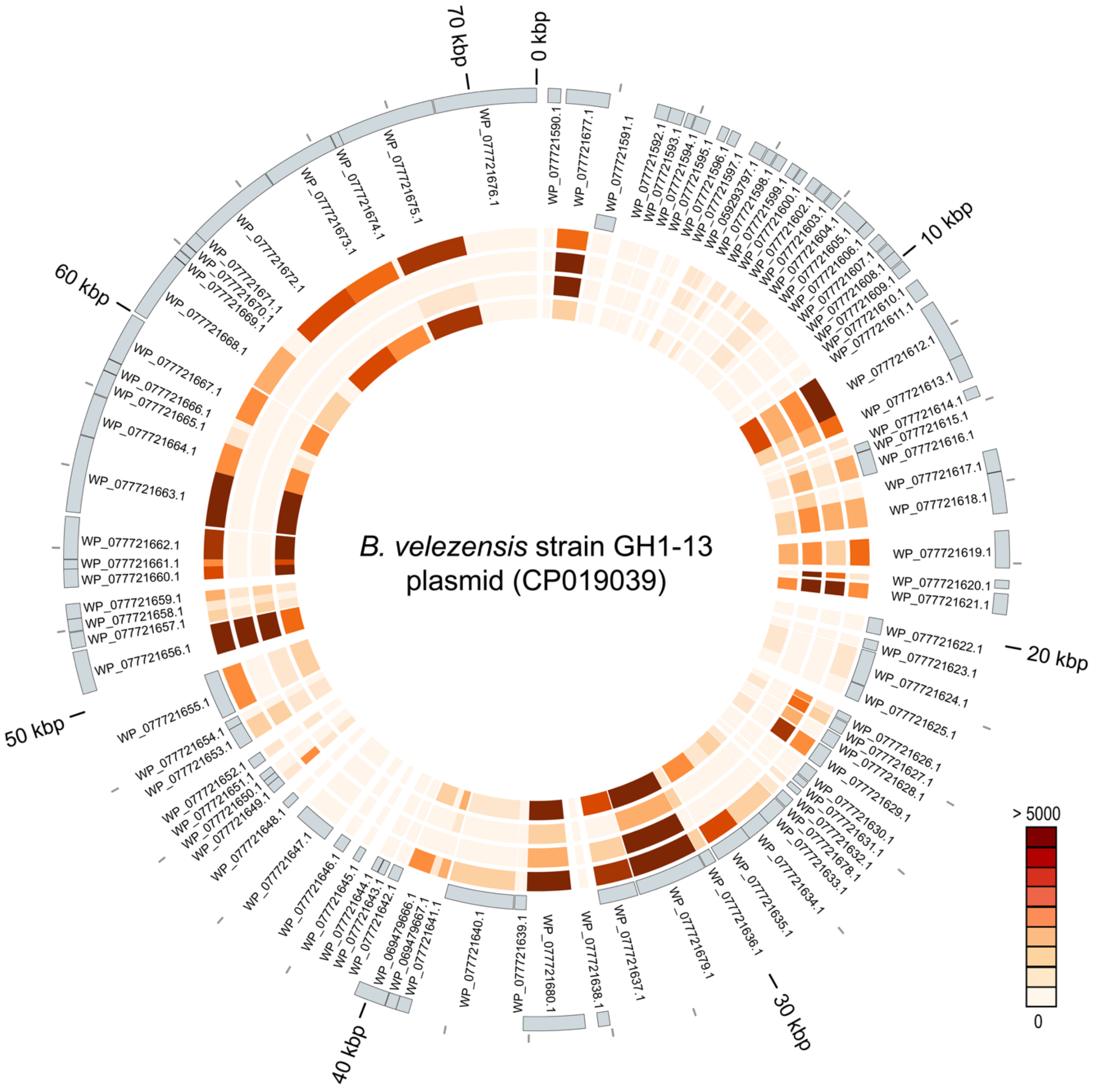

Supplementary Figure 3. Growth curves of wild-type and mutant strains GH1-13 and FZB42 on various single carbon sources.

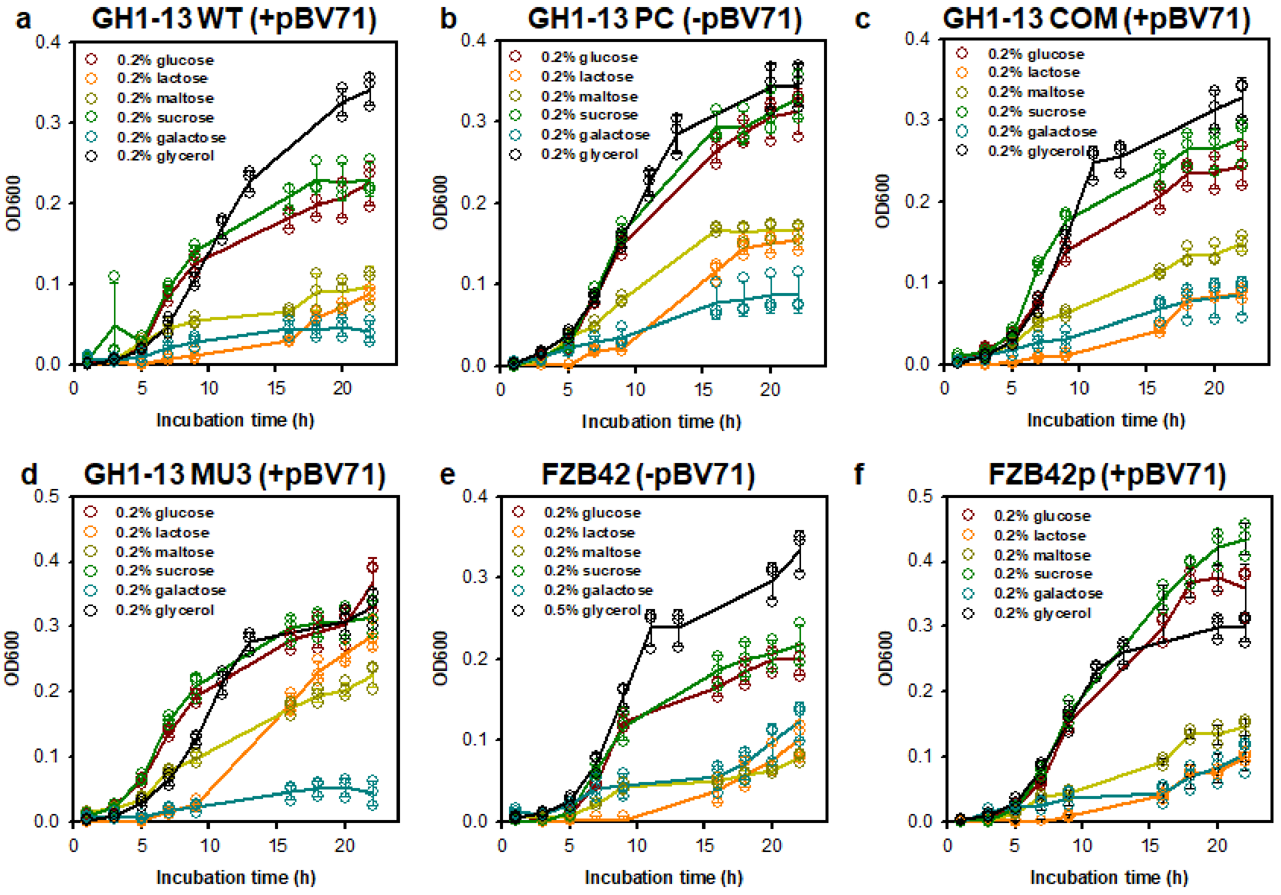

a Wild-type (WT) strain GH1-13. **b** Plasmid-cured (PC) variant of strain GH1-13. **c** Reverse complement (COM) of PC cells with pBV71. **d** Mucoid variant (MU3) of strain GH1-13 with pBV71. **e** Wild-type strain FZB42 with no plasmid. **f** Transconjugant FZB42p with plasmid pBV71. Cells exponentially grown in tryptic soy broth (TSB) at 25 °C with aeration (200 rpm) were harvested by centrifugation at 3,515 ×*g* for 5 min, washed with sterile M9 medium, centrifuged again, and suspended at an initial optical density of 0.001 at a wavelength of 600 nm (OD600) in the M9 culture media, supplemented with 0.2% (w/v) glucose, lactose, maltose, sucrose, galactose, and glycerol as sole carbon and energy sources. When cultivated at 25 °C with aeration (200 rpm), OD600 was timely measured and results from three times independent cultures are reported as mean with standard deviation (error bar) on each curve. When comparing growth curves between carbon sources, significant differences between the non-mucoid phenotypes (WT, COM, FZB42, and FZB42p) and the mucoid phenotypes (MU3 and PC) of strains GH1-13 and FZB42 on lactose were observed (two-tailed *t*-tests, $P < 0.05$), whereas insignificant changes were observed among strains in glucose, as shown in Fig. 5**a**–**b** in the main text. Source data of figures **a**–**f** are shown in Supplementary Data 3.

Supplementary Figure 4. RT-PCR results of 14 Rap homologous loci in pBV71 and chromosome.

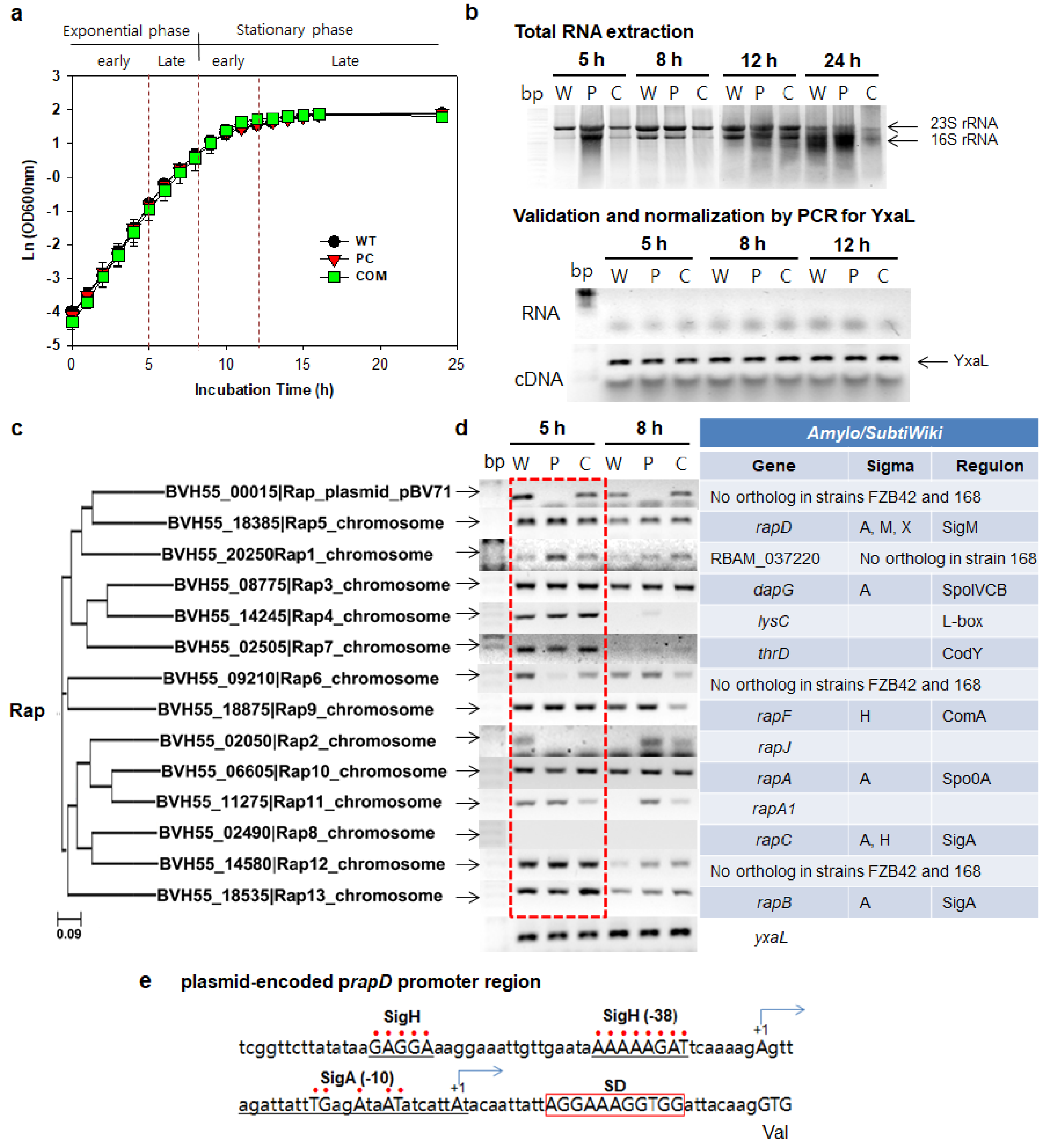

a Semi-logarithmic growth curves from triplicate cultures of each strain in tryptic soy broth at 25 °C with shaking (180 rpm). Incubation periods during early exponential (5 h), late exponential (8 h), early stationary (12 h), and late stationary (24 h) phases were determined by changes in growth rate (slope). **b** Extraction and validation of total RNA and construction of cDNA from different phases. The 16S and 23S ribosomal RNA (rRNA) bands were detected by gel electrophoresis (upper), removal of DNA was verified by a control PCR using primers of the *yxaL* gene directly on RNA extracts (middle panel),

and the cDNA levels for quantitative PCR were normalized with the *yxaL* gene (lower panel). **c** A phylogenetic tree from a Clustal alignment of the 14 Rap homologous DNA sequences. **d** Semi-quantitative PCR bands showing expression of 13 Rap homologous genes, but not of *rapC* gene (BVH55_02490). Gene name (locus), sigma factors, and transcriptional regulators of the annotated genes in *Bacillus subtilis* strain 168 (*SubtiWiki*) and *B. velezensis* strain FZB42 (*AmyloWiki*) are shown in the table. **e** Recognition sites of sigma factors A and H from -10 and -38 upstream regions of the stringent Shine-Dalgarno (SD) sequence in the pBV71 plasmid p*rapD* promoter.

Supplementary Figure 5. RT-PCR results of wild-type (W) pBV71 plasmid-containing, plasmid-cured (P), and complemented (C) cells of *Bacillus velezensis* strain GH1-13 for genes involved in the regulatory network for conjugation, competence, biofilm formation, catabolite control, cell wall metabolism, and sporulation during early (5 h) and late (8 h) exponential phases.

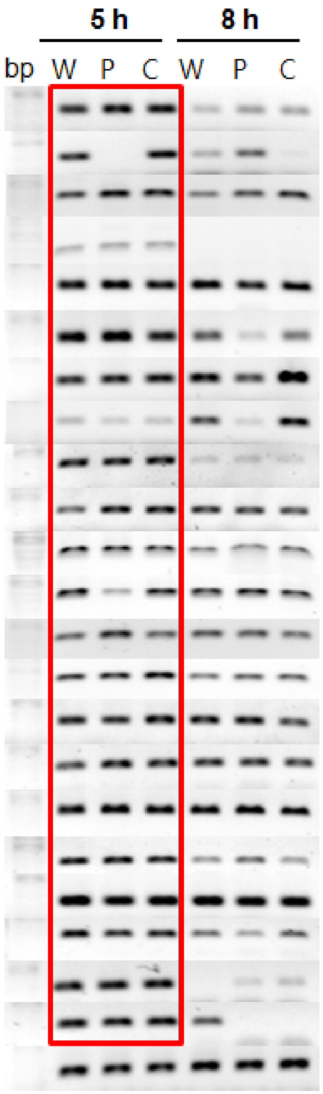

Amylo/SubtiWiki			
**Gene**	**Sigma**	**Regulon**	**Function**
kinC	A	Spo0A	Sensor histidine kinase for sporulation
spo0F	A, H	Spo0A	Phosphorelay for Spo0A activation
bceS			Bacitracin sensor histidine kinase
bceR			Response regulator for resistance to toxic peptides
liaS	A	SigA	Sensor histidine kinase LiaS
liaR	A	SigA	Response regulator LiaR
liaH	A	SigA	Stress response protein LiaH for LiaR activation
liaI	A	SigA	Stress response protein LiaI for LiaR activation
yhcY	A	SigA	Sensor histidine kinase for LiaR activation
yhcZ	A	SigA	Response regulator for LiaR activation
degS	A	SigA	Sensor histidine kinase
degU	A	SigA	Response regulator for biofilm formation
phoR	A, B, E	SigA	PhoP activation for additional metabolic pathways
phoP	A, B, E	SigA	Response regulator for additional metabolic pathways
resE	A	ResD	Cell wall metabolism sensor histidine kinase
resD	A	ResD	Response regulator for PhoP activation
lytS			Sensor histidine kinase for autolysis
lytT			Response regulator for autolysis
abrB	AbrB		Transcriptional factor during transition
slrR		Abh	Transcriptional regulator for biofilm
lacR1			Polysaccharide utilization
glvR	A	SigA	Maltose utilization
yxaL			

Supplementary Figure 6. Typical photos of *in vitro* antagonistic tests of wild-type (WT), plasmid-cured (PC), and complemented (COM) cells of strain GH1-13 against soil-borne plant pathogens. Strain GH1-13 cells were exponentially cultivated to an OD600 of 1.0 at 600 nm in tryptic soy broth (TSB) at 25 °C and 180 rpm and 5 μL volume of the culture media from each strain was inoculated into a sterile 3M filter paper (diameter, 6 mm). Three bacterial disks and one control disk (Ctl) soaked only with 5 μL TSB were placed on the four corners with the same distance kept in 4 fungal cultures (*R. solani*, *C. acutatum*, *F. solani*, and *G. moniliformis*) and 2 bacterial cultures (*P. carotovorum* and *S. acidiscabies*) inoculated respectively onto the centers of potato dextrose agar plates and on the lawns of TSA plates. After incubation for appropriate times as indicated by the inhibition zone against test plant pathogens, the inhibitory zone of a tested plant pathogen around the bacterial disk and the no inhibition zone from control disk were measured from the disk center with a scale in mm, as reported in Fig. 7a in the main text. Data of the figure are shown in Supplementary Data 3.

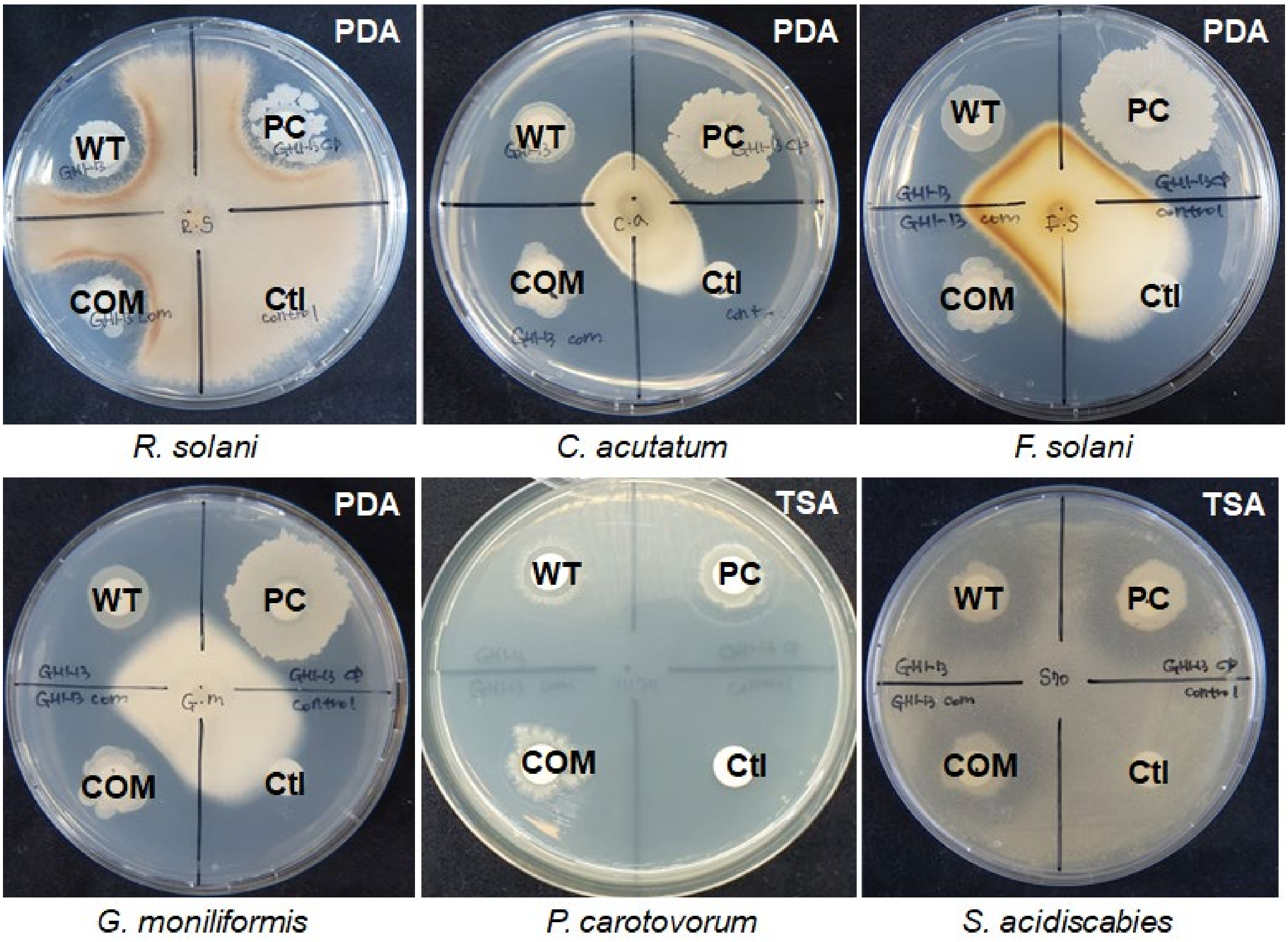

Supplementary Figure 7. Analysis of macrolactins and surfactins by high performance liquid chromatography coupled with UV spectroscopy and mass spectrometry. The sample was prepared with 10 mL of filtered culture fluid of *Bacillus velezensis* strain GH1-13 by Sep-Pak C18 solid-phase extraction of lipopeptides and polyketides, as described in the Methods section.

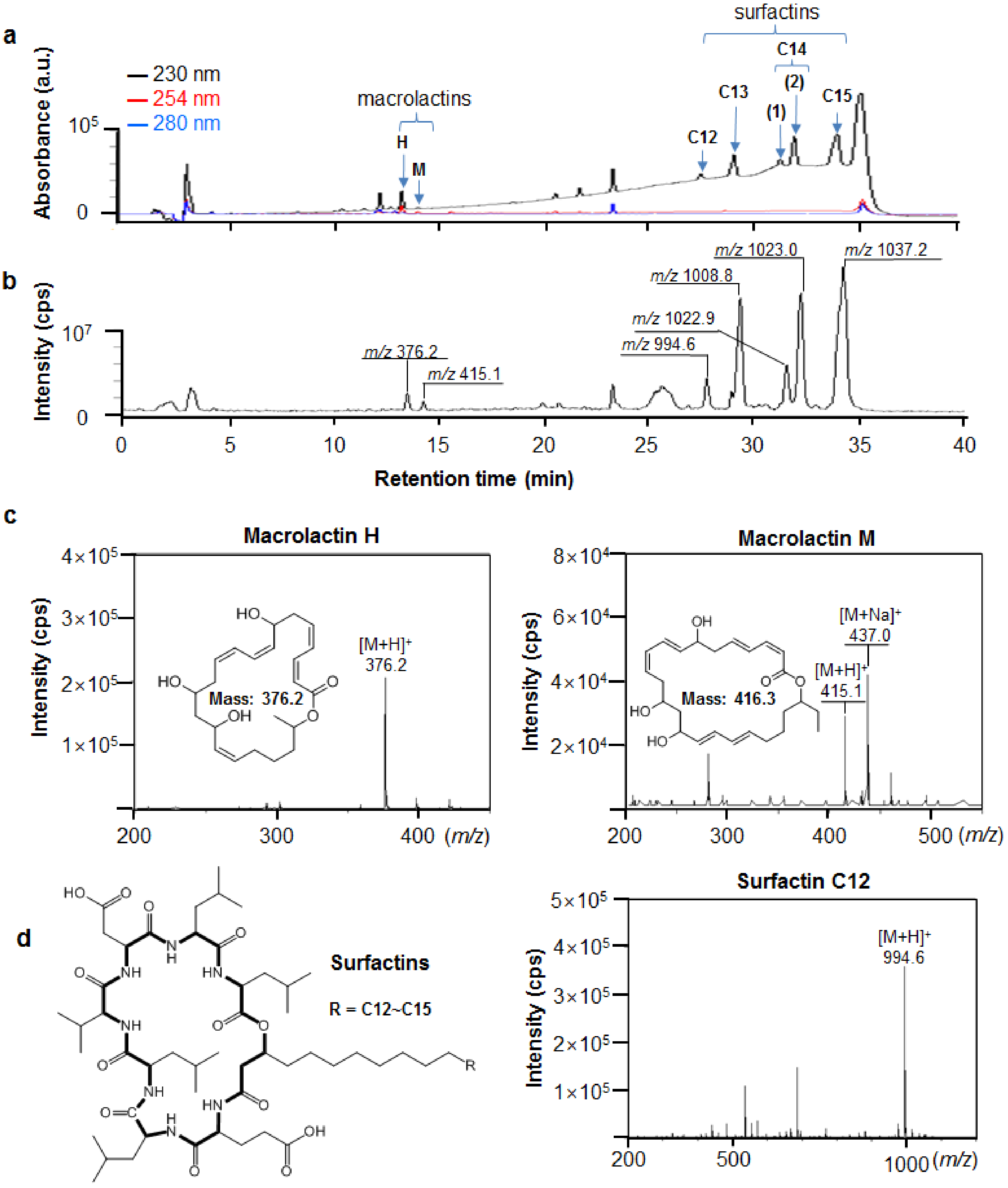

(Supplementary Figure 7, *continued*)

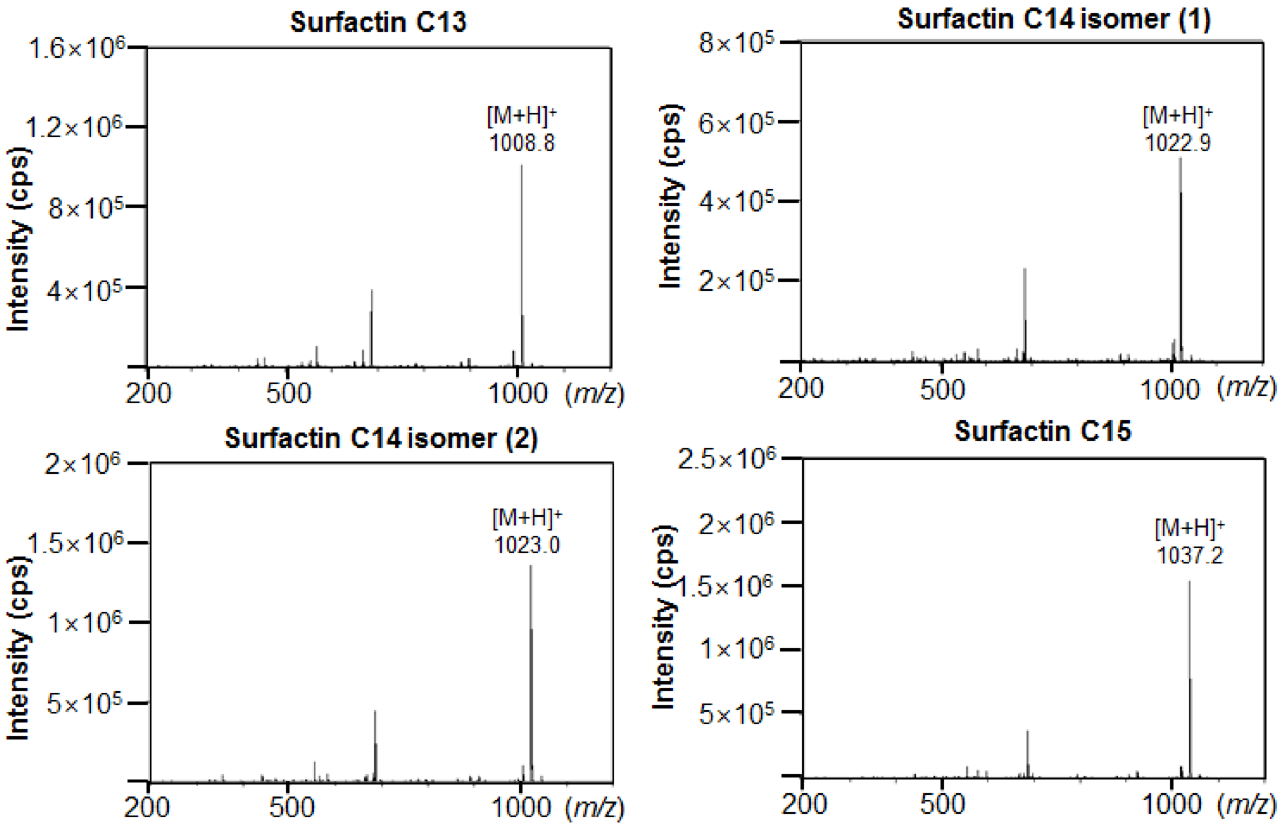

a Chromatographic peaks of macrolactins (H and M) and surfactins (C12 to C15) detected by UV spectroscopy at the indicated wavelengths of 230, 254, and 280 nm. **b** The base peak mass chromatogram showing the assigned molecular ion $[M+H]^+$ peaks of macrolactins (H and M) and surfactins (C12 to C15). **c** Mass spectra and chemical structures of macrolactin H and macrolactin M at the peaks of *m/z* 376.2 and *m/z* 415.1. **d** Mass spectra and chemical structures of surfactin variants: C12 (*m/z* 994.6), C13 (*m/z* 1008.8), C14 isomer 1 (*m/z* 1022.9), C14 isomer 2 (*m/z* 1023.0), and C15 (*m/z* 1037.2). Percentages of the individual peak areas in the mass spectrometry of macrolactins and surfactins are shown in Supplementary Data 3.

Supplementary Figure 8. Transcriptome analysis of wild-type (WT) pBV71 plasmid-containing and plasmid-cured (PC) cells of *Bacillus velezensis* strain GH1-13. Cells were harvested at different incubation times of 5, 8, 12, and 24 h during cultivation in tryptic soy broth at 25 °C with shaking (180 rpm).

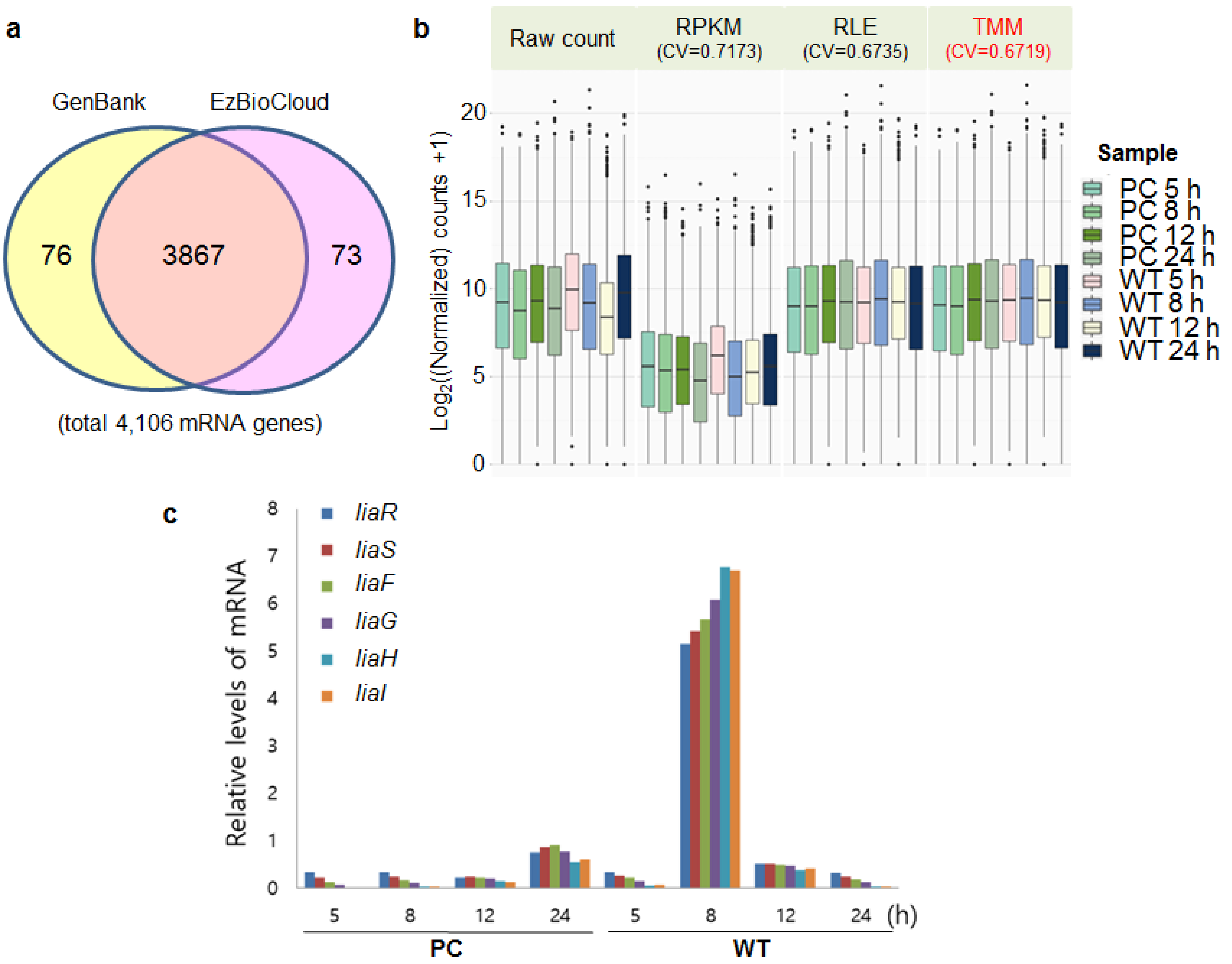

a A Venn-Diagram showing a total of 4,106 genes obtained from the two genome annotation data of *B. velezensis* strain GH1-13 in the NCBI GenBank and ChunLab's EzBioCloud, respectively, including 76 and 73 unique candidate genes. The 118 non-coding RNA genes that do not encode proteins were excluded from the transcriptome analysis. After removing low-quality reads with low average coverage (less than 100 reads) and pBV71 plasmid genes, RNA-Seq reads of 3,263 genes in the chromosome were normalized and used for statistical analysis of four different growth phases of the two strains. **b** Normalization of RNA-Seq raw counts using RPKM, RLE, and TMM methods. The TMM normalization method which gave the lowest coefficient of variation (CV) was used in this study. **c** Relative expression levels of the *liaIHGFSR* genes in the different phases of WT and PC cells. A significant difference in the gene expression pattern of *liaIHGFSR* operon was determined between the different phases of the two strains by the analysis of variance (*F*-test, cutoff $P < 0.01$) and Yates-corrected chi-square tests (χ^2-test, cutoff $P < 0.01$), as described in the main text.

Supplementary Figure 9. Pearson correlation analysis with a bivariate-normal distribution of the $\log_2$ average normalized values of secretome, proteome, and transcriptome data. The $\log_2$ average normalized values of secretome and proteome in Supplementary Data 4 and 6 were computed by dividing the NSAF value with the mean of each column. The $\log_2$-transformed values of NSAF per mean and TMM normalized RNA-Seq counts in Supplementary Data 5 were subtracted by the new mean to make the sum equal to zero. Pearson correlation coefficients between the experiment data are given in parentheses.

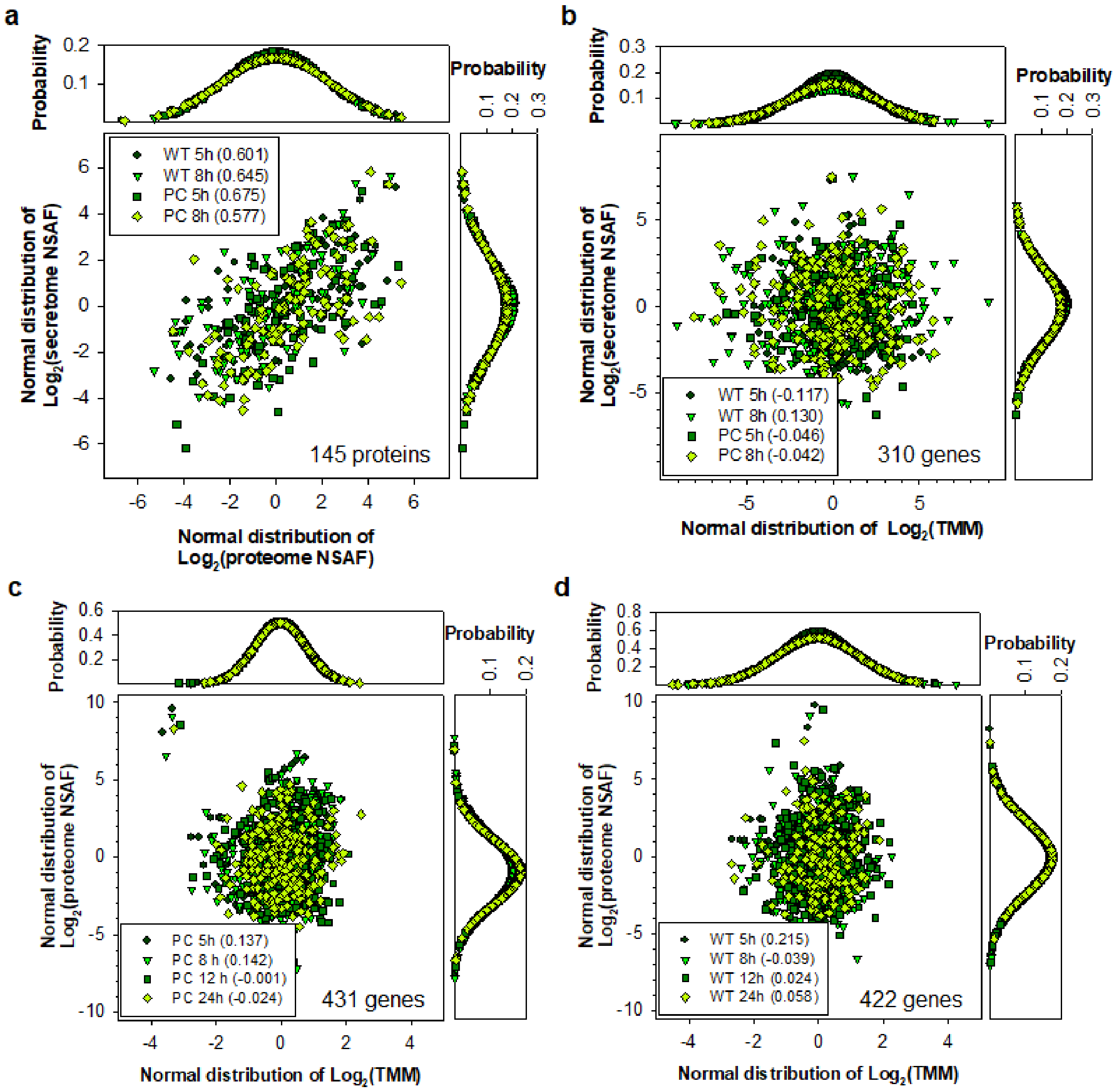

a-b Correlation coefficients of secretome with the corresponding proteome (**a**) and transcriptome (**b**) in early (5 h) and late (8 h) exponential phases of WT and PC cells. **c-d** Correlation coefficients between proteome and transcriptome data at different growth phases (5, 8, 12, and 24 h) of PC cells (**c**) and WT cells (**d**).

Supplementary Figure 10. A confidence view of the functional association network analysis. The STRING analysis shows top-ranked pathways of the genes differentially expressed and co-expressed, as listed in Supplementary Data 5, in response to the plasmid-induced *liaIH* expression in the late exponential phase of *Bacillus velezensis* strain GH1-13.

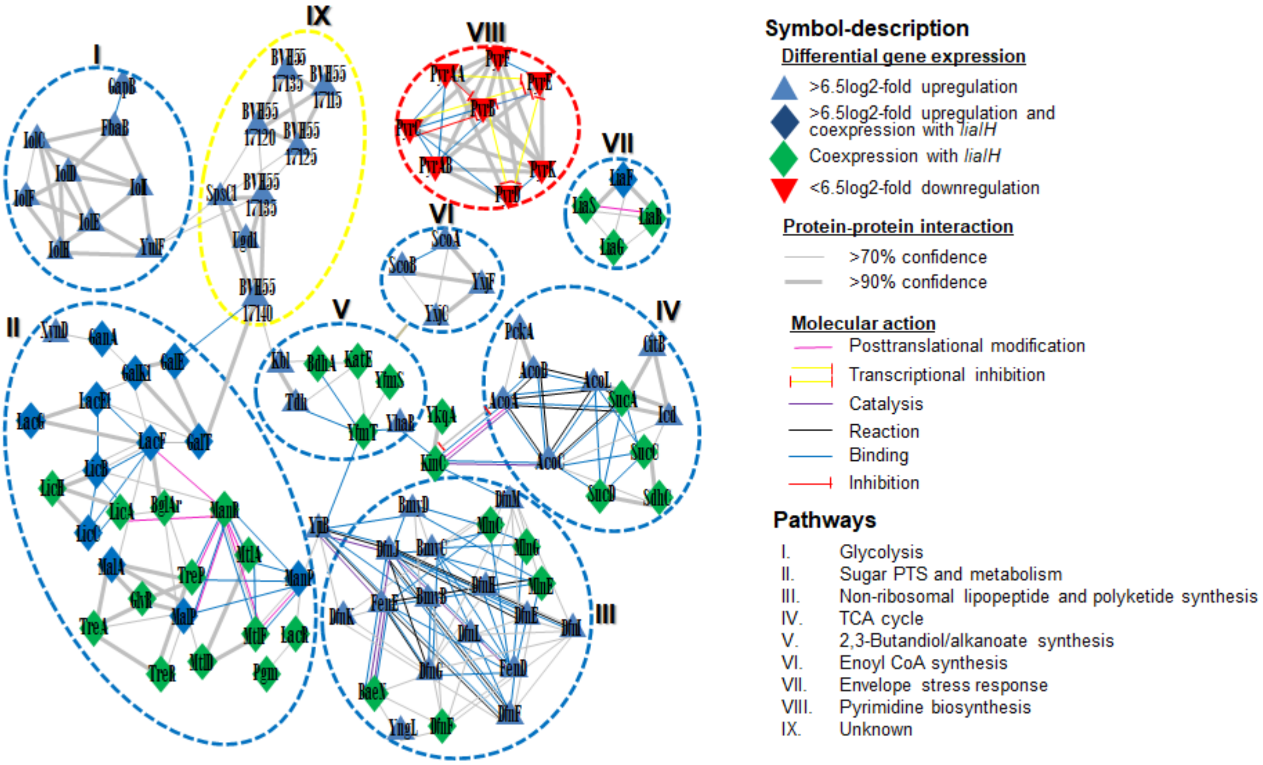

Supplementary Figure 11. *In-gel* activity assays for NAD(P)-dependent dehydrogenases. Whole cell extracts (20 µg protein per lane) were obtained from early (5 h) and late (8 h) exponential phases of wild-type (WT) pBV71 plasmid-containing and plasmid-cured (PC) cells of *Bacillus velezensis* strain GH1-13 during cultivation in tryptic soy broth at 25 °C with aeration (180 rpm). Non-denaturing blue native polyacrylamide gel electrophoresis was performed, as described in the Methods section in-gel activity assays. Panels on the right show relative gene expression levels of each gene in the transcriptome data for different incubation times in two strains. Gene expression pattern in late (8 h) exponential phase is highlighted with a red dotted line box.

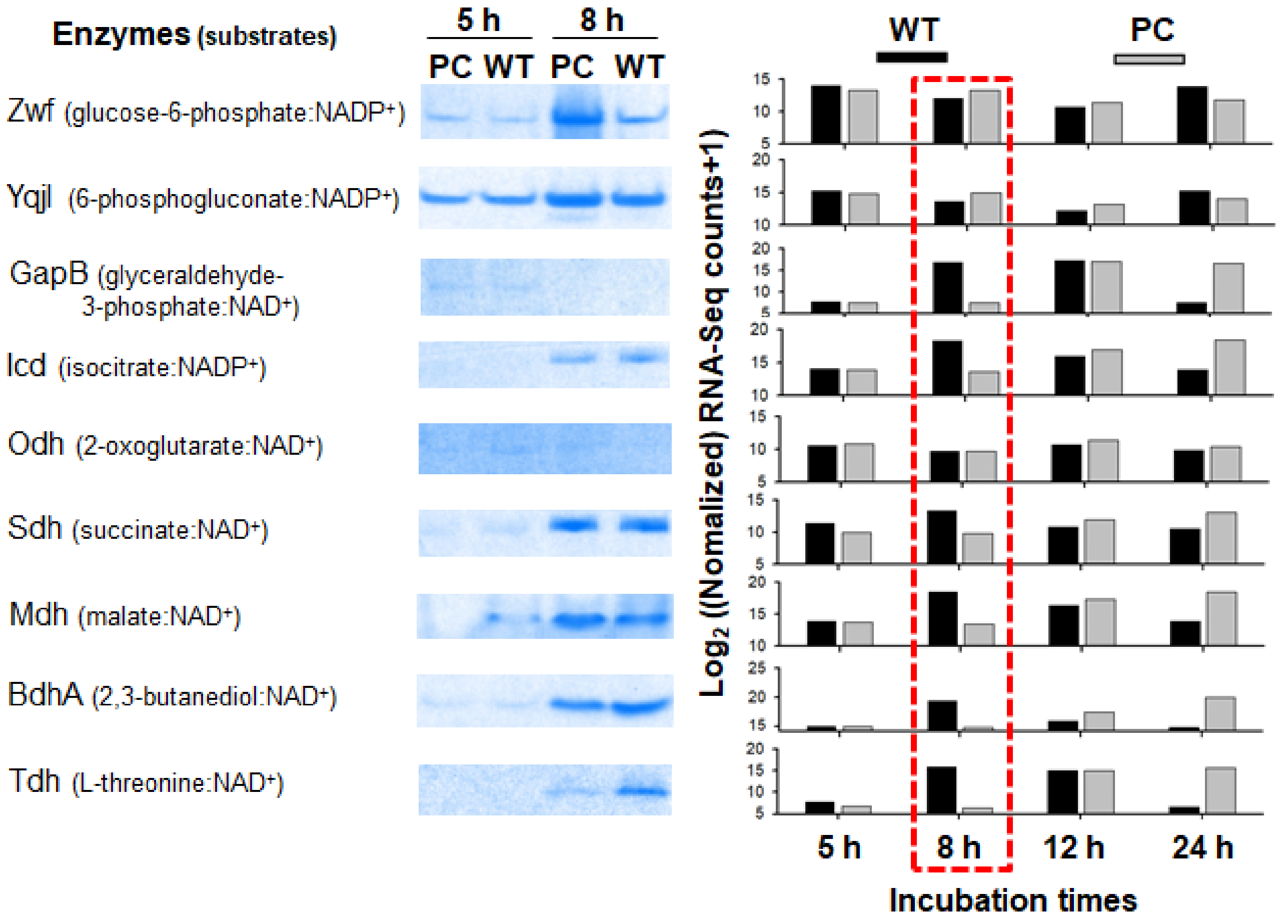

Supplementary Figure 12. Original images of gels and western blots as well as blue native gels for the *in-gel* activity staining of NAD(P)-dependent dehydrogenases, which are respectively shown in Fig. 5c, Fig. 8d, and Fig. 10 in the main text.

Fig. 5c

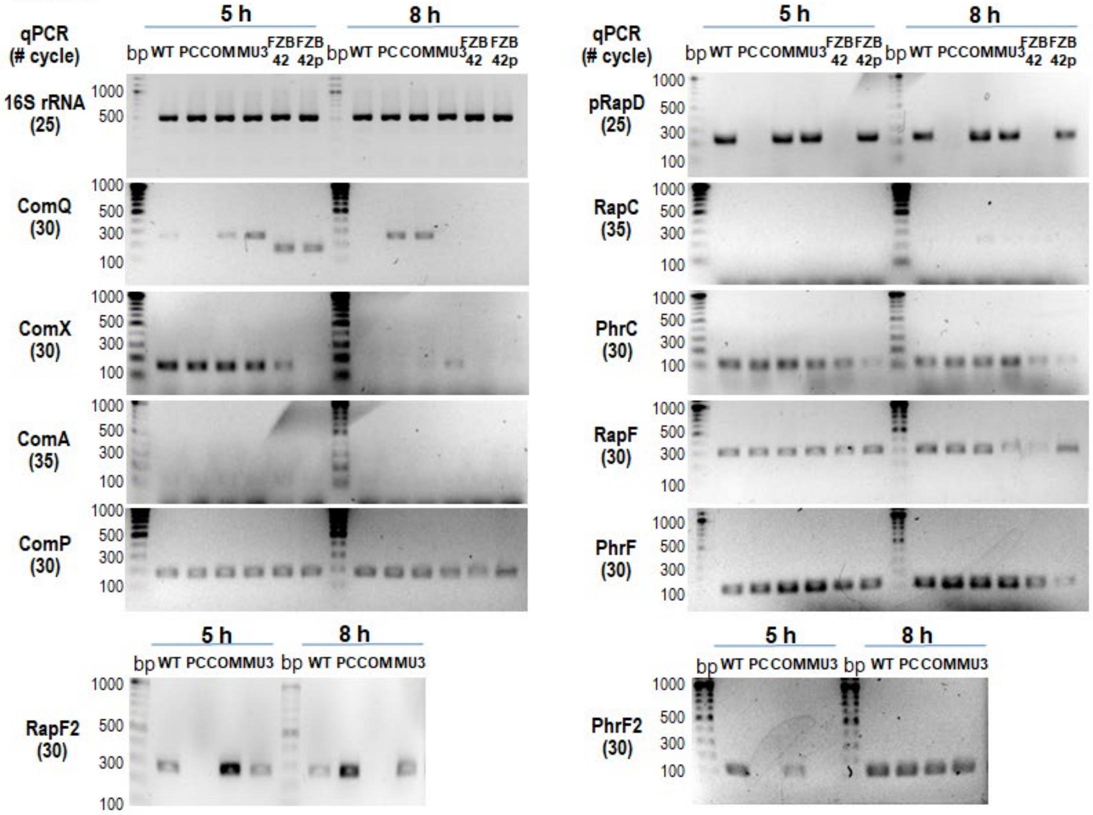

Fig. 8d

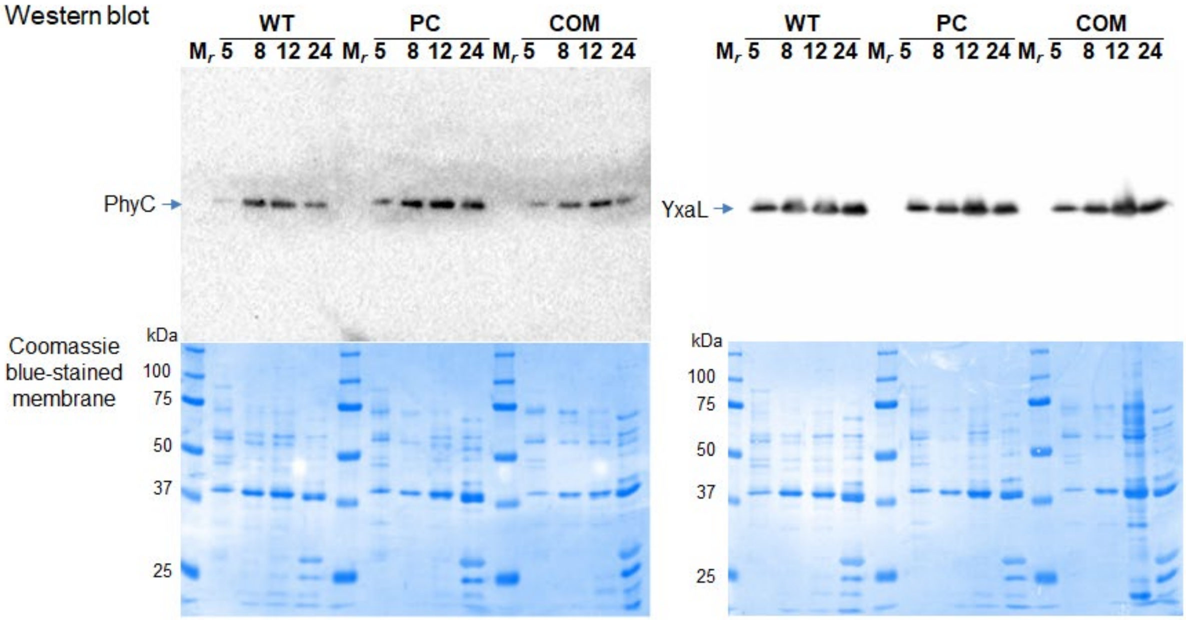

(Supplementary Figure 12, *continued*)

Fig. 10

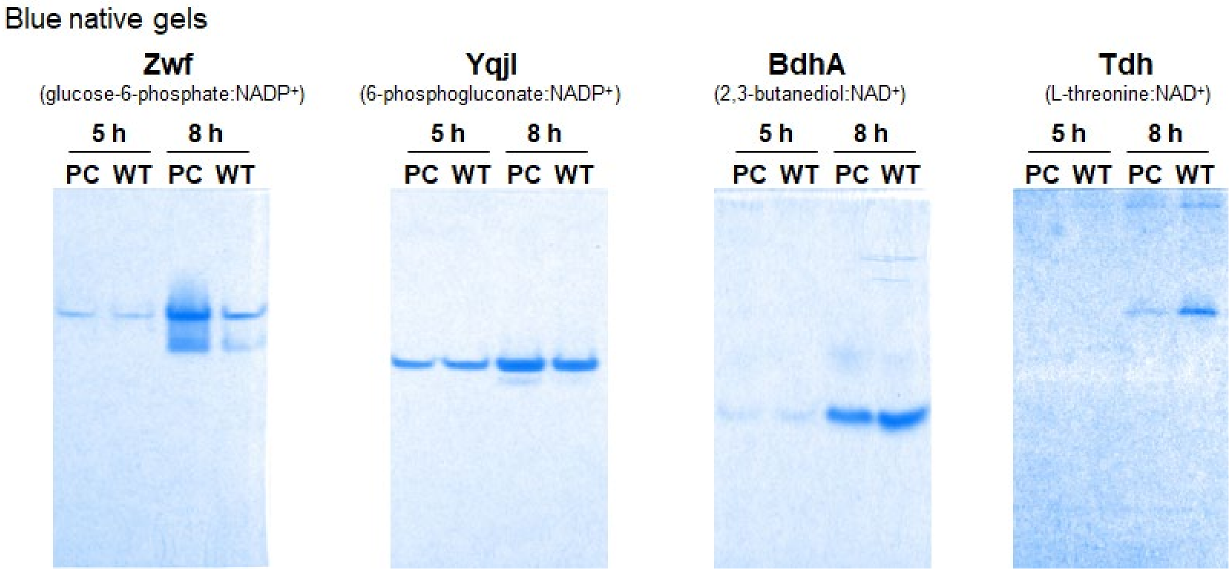

Supplementary Table 1. List of PCR primer sets used for the detection of full-length pBV71 plasmid.

(#)	PCR region	Primer names	Nucleotide sequences: 5' to 3'
(1)	-24 to 3920	pBV-F(-24)	5'-CAGAAGACCCATTTAAAGGG
		pBV-R3920	5'-ATCTCTCCATTCTCAAAATCT
(2)	3921 to 7910	pBV-F3921	5'-CAAGGGTGGAGCTTAGT
		pBV-R7910	5'-TCTTAGCCCAACCTAATGA
(3)	7911 to 11845	pBV-F7911	5'-GAAAGTGTTGGGAGTTAGT
		pBV-R11845	5'- AGTCTGACTGTATAGACTTCT
(4)	11846 to 15680	pBV-F11846	5'-CTGCGTGGAGAATTGATT
		pBV-R15680	5'-ATCGAGGCTGTCCTAAG
(5)	15681 to 19390	pBV-F15681	5'-ACAGCGAGCATGATTTAG
		pBV-R19390	5'-CCTTCTTAATTGAATCTTCGAT
(6)	19391 to 23435	pBV-F19391	5'-GGTCTGAAAAAGACCCTT
		pBV-R23435	5'-GGGAGTTAAGTGATTAAGGT
(7)	23436 to 27160	pBV-F23436	5'-CCCTTTGGTTGATAGAGTT
		pBV-R27160	5'-GCAATTTGCAATAGATGCTA
(8)	27161 to 31150	pBV-F27161	5'-CGGAATGGTTCTTCCGA
		pBV-R31150	5'-AGGGTTTGCCAGTAAGA
(9)	31151 to 33635	pBV-F31151	5'-TTGATGCGTTGTCAGTG
		pBV-R33635	5'-AGGGGGTGGATATGCT
(10)	33636 to 36688	pBV-F33636	5'-ACAGCCCCAACAATGA
		pBV-R36688	5'-TCGATATAATTGGGAAAGCTG
(11)	36689 to 40081	pBV-F36689	5'-ACAGAGAAGTTGCAACAC
		pBV-R40081	5'-AGAGATTGGTTTTGCTGAAT
(12)	40082 to 42768	pBV-F40082	5'-AATGAGTTCTGTGAGTGATT
		pBV-R42768	5'-ACAAGAGAGCAGAGTGT
(13)	42769 to 46550	pBV-F42769	5'-TTGTAAAACTTGGGTATCTAAATC
		pBV-R46550	5'-CCACTTATGAACCCATTTATT
(14)	46551 to 50330	pBV-F46551	5'-GTTATCATCCTCTAACACGA
		pBV-R50330	5'- TTAGTATACATTCGCAATTAGATT
(15)	50331 to 54180	pBV-F50331	5'-ATAAGTGTTGACTATACATCGA
		pBV-R54180	5'-TGTCATCTAAAGTTTCATAACAA
(16)	54181 to 58170	pBV-F54181	5'-GATACGTTAAAGTTATTAAAGTTTC
		pBV-R58170	5'-CACTTTATCAGTCATGTATTTTTTAG
(17)	58171 to 62090	pBV-F58171	5'-TGGATAAGTTAAAGGGTTCA
		pBV-R62090	5'-GGTAACCCTTTTAAAGTAAGG
(18)	62091 to 65912	pBV-F62091	5'-AACTCATTAAGGGAGAATCAA
		pBV-R65912	5'-AGTCCATGAGTGAGTAGTT
(19)	65913 to 69790	pBV-F65913	5'-CATGTATTTCGAAGAATTTCAAC
		pBV-R69790	5'-GGGTTGAACCCCCAA
(20)	69791 to +420	pBV-F69791	5'-GATGGCATTTGTAAAGAATGA
		pBV-R420	5'- ACCCCCAAAGCAACA

Notes. The primer sets were designed to amplify their target sequences in a head-to-tail arrangement on the genome of pBV71 (NCBI accession number CP019039.1). Numbers (#) of primer sets are identical to those in the main text and figures.

Supplementary Table 2. Minimum inhibitory concentrations (MIC) of antimicrobial agents active aganist non-mucoid (WT), mucoid variants with (MU3) or without (PC) pBV71, reverse complement (COM), and trans-conjugant (FZB42/pBV71) of *Bacillus velezensis* strains GH1-13 and FZB42.

Strains	GH1-13				FZB42	
Antibiotics (units)	WT	MU3	PC	COM	WT	pBV71
Amikacin (μg/mL)	4	2	16	4-32	4	8
Ampicillin (μg/mL)	128	**16**	128	32-64	16-64	16
Antimycin A (μg/mL)	>128	>128	>128	>128	>128	>128
Bacitracin (U/mL)	>10	**0.63-1.25**	**0.63-1.25**	5-10	>10	>10
Carbenicillin (μg/mL)	128	>128	>128	>128	>128	>128
Chloramphenicol (μg/mL)	2	<0.06-0.25	16-32	2-8	4	8-16
Ciprofloxacin (μg/mL)	1	0.25-0.5	0.5	<0.06	0.5-1	0.25-0.5
Cephalexin (μg/mL)	0.25	0.25-0.5	0.25	0.25	0.1-0.5	1-4
Colistin (μg/mL)	>64	64	>64	>64	>64	>64
Doxycycline (μg/mL)	1-2	0.5	0.5	0.5	0.25-0.5	<0.06
Erythromycin A (μg/mL)	0.1	<0.06	0.1-0.25	0.1	0.1	0.1
Gramicidin (μg/mL)	>128	**0.25-0.5**	**4**	32-128	**32**	>128
Gentamicin (μg/mL)	2	1-2	4	1	2	0.5-1
Hygromycin (μg/mL)	128	32-64	>128	128	128	128
Kanamycin (μg/mL)	4-16	2	8	4	8	2
Mitomycin C (μg/mL)	0.08-0.16	0.08-0.16	0.16	0.08	0.16-0.31	0.31
Nalidixic acid (μg/mL)	1	4-8	8	4	1	2-4
Neomycin (μg/mL)	2	2-4	8	4	2	2
Norfloxacin (μg/mL)	1	1	0.25	1	0.1	0.25
Oxolinic acid (μg/mL)	0.5-1	0.5	0.5-2	0.5	1-2	0.5
Oxytetracycline (μg/mL)	16-32	16-32	16-32	16	1	0.1-0.5
Penicillin G (U/mL)	>100	>100	>100	>100	>100	>100
Piromidic acid (μg/mL)	2-8	2-4	1-4	2	0.25-2	1-2
Polymyxin B (μg/mL)	32-64	32-64	64	64	32	32
Rifampin (μg/mL)	0.25-0.5	<0.06	0.1-0.25	0.1-0.25	0.25-0.5	0.1-2
Spectinomycin (μg/mL)	64->128	64	128	64	64	64
Streptomycin (μg/mL)	32-64	8-16	128	32-64	16	8
Sulfamethazine (μg/mL)	>128	>128	>128	>128	>128	>128
Tetracycline (μg/mL)	4-8	8	4-16	8	0.1-0.5	<0.06
Vancomycin (128 μg/mL)	0.25-0.5	0.1	0.25	0.25	0.5	0.1
Lysozyme (U/mL)	>10,000	>10,000	>10,000	>10,000	>10,000	>10,000
Sodium selenite (mg/mL)	5	**2.5**	**2.5**	5	5	5
Potassium tellurite (μg/mL)	31-62.5	**8**	**2-4**	31	**8**	31

Notes. Antimicrobial susceptibility test to determine the range of MIC resulting from at least five repeated tests for each isolate. The MIC was determined after 24 h incubation at 25 ºC with aeration (180 rpm) in 96-well microtiter plates loaded with 2-fold serial dilutions of each agent in 100 μL tryptic soy broth (TSB), inoculated by ~10^4 colony-forming units exponentially grown in TSB medium at 25 ºC and 180 rpm. Red-colored MIC values (ranges) indicate significant differences (two-tailed *t*-tests, $P < 0.05$) as compared to the WT strain GH1-13.

Supplementary Table 3. API 50 CH test results for anaerobic carbohydrate fermentation of pBV71 plasmid-containing and plasmid-free strains of *Bacillus velezensis* GH1-13 and FZB42.

Strains	GH1-13				FZB42	
	WT	MU3	PC	COM	WT	pBV71
Plasmid pBV71	+	+	-	+	-	+
Glycerol	-V	++	++	++	-V	++
Erythritol	--	--	--	--	--	--
D-Arabinose	--	--	--	--	--	--
L-Arabinose	++	++	++	++	-+	++
Ribose	++	++	++	-+	++	++
D-Xylose	-V	++	-V	V+	V+	V+
L-Xylose	--	--	--	--	--	--
Adonitol	--	--	--	--	--	--
β-Methyl-xyloside	--	--	--	--	--	--
Galactose	-V	-V	-+	-V	-V	-V
D-Glucose	++	++	++	V+	++	++
D-Fructose	++	++	++	++	++	++
D-Mannose	++	++	++	V+	++	++
L-Sorbose	--	--	--	--	--	--
Rhamnose	--	--	--	--	--	--
Dulcitol	--	--	--	--	--	--
Inositol	++	++	++	++	-V	++
Mannitol	++	++	++	++	-V	++
Sorbitol	-+	++	++	-+	-V	-+
α-Methyl-D-mannoside	--	--	--	--	--	--
α-Methyl-D-glucoside	-V	V+	++	-+	-+	++
N-Acetyl glucosamine	--	--	--	--	--	--
Amygdalin	--	--	--	--	--	--
Arbutin	-V	-V	++	V+	-+	V+
Esculin	++	++	++	++	++	++
Salicin	-V	-+	-+	-+	++	++
Cellobiose	++	++	++	++	++	++
Maltose	-V	-+	-V	-+	-V	-+
Lactose	-V	-+	-+	-V	-V	-V
Melibiose	--	--	--	--	--	--
Sucrose	++	++	++	++	++	++
Trehalose	-V	V+	-+	-V	++	++
Inulin	--	--	--	--	--	--
Melezitose	--	--	--	--	--	--
D-Raffinose	++	++	++	-V	-V	++
Starch	-V	-V	++	++	-V	++
Glycogen	-V	V+	-+	++	-V	++
Xylitol	--	--	--	--	--	--
β-Gentibiose	-V	V+	-+	-V	-V	-V
D-Turanose						

D-Lyxose	--	--	--	--	--	--
D-Tagatose	--	--	V+	--	--	--
D-Fucose	--	--	--	--	--	--
L-Fucose	--	--	--	--	--	--
D-Arabitol	--	--	--	--	--	--
L-Arabitol	--	--	--	--	--	--
Gluconate	--	--	--	--	--	--
2-Keto-gluconate	--	--	--	--	--	--
5-Keto-gluconate	--	--	--	--	--	--

Notes: API 50CH test (BioMérieux, Salt Lake City, UT) was performed using API 50 CHB/E media inoculated with exponentially growing cells at an OD600 of 0.05, incubated under mineral oil at 25 ºC. The carbohydrate fermentation reaction in each tube was recorded after 24 h (first symbol) and 48 h (second symbol) of incubation according to manufacturer's manual: -, negative, V, variable; +, positive.

Supplementary Table 4. List of PCR primer pairs for the analysis of regulatory gene expression patterns in *Bacillus velezensis* strains GH1-13 and FZB42.

GenBank locus tag		Description	Primer pairs
GH1-13	FZB42		Name: 5' to 3' nucleotide sequence
BVH55_00015		Aspartate phosphatase (plasmid)	pRap-F241: 5'-GCGTTAAGAACACCTACAGA<br>pRap-R476: 5'-AATGCATTCTCAGCGTGG
BVH55_00285		MobA/MobL family (plasmid)	TraA-F347: 5'-ACCTTCACATGCTCCTGA<br>TraA-R539: 5'-TGTATTGAAACGTCCGCAAC
BVH55_00430		Hypothetical protein (plasmid)	TraL-F93: 5'-TGCGGCTGTAAAAGATGGA<br>TraL-R379: 5'-GGCAGGTTACATCAATCCA
BVH55_20250	RBAM_037220	Aspartate phosphatase, Rap1 (chromosome)	Rap1-F13: 5'-ATGGCCCACGAAACAGTT<br>Rap1-R308: 5'-AACTCGTGCATTCCGGAA
BVH55_02505	RBAM_003080	Aspartate kinase, Rap2 (chromosome)	Rap2-F80: 5'-ATCCCTCGCGGAAAGC<br>Rap2-R331: 5'-ACTGATCGGGATTGGCTT
BVH55_08775	RBAM_016600	Aspartate kinase, Rap3 (chromosome)	Rap3-F119: 5'-TATCGGCGATGGGAAGAA<br>Rap3-R384: 5'-AGCGAAAAGCCGCTCT
BVH55_14245	RBAM_025540	Aspartate phosphatase, Rap4 (chromosome)	Rap4-F96: 5'-AGGCCATGATGTCGT<br>Rap4-R406: 5'-AACCGGCAACGACTGT
BVH55_18385	RBAM_033580	Aspartate phosphatase, Rap5 (chromosome)	Rap5-F173: 5'-CACGGTTTCAGCTGCTGT<br>Rap5-R440: 5'-GCAGGCGACTTCATCATGT
BVH55_09210	No homolog	Aspartate phosphatase, Rap6 (chromosome)	Rap6-F79: 5'-TGGGTCGGTAGAGCAGA<br>Rap6-R355: 5'-GGGAGAATGCCAACTCCT
BVH55_02050	RBAM_004040	Aspartate phosphatase, Rap7 (chromosome)	Rap7-F85: 5'-GCTGAAGCGCTGAAGC<br>Rap7-R290: 5'-AGTAAGCCGGTCAGCTT
BVH55_02490	RBAM_004010	Aspartate phosphatase, Rap8 (chromosome)	Rap8-F25: 5'-TCAGCAGTCGGGCAGA<br>Rap8-R293: 5'-CCTTTGAGGCCGGCCT
BVH55_18875	RBAM_034530	Aspartate phosphatase, Rap9 (chromosome)	Rap9-F143: 5'-ACCTCGAACCGCTGGA<br>Rap9-R449: 5'-TAAGCCTGCCGGGCAT
BVH55_06605	RBAM_012450	Aspartate phosphatase, Rap10 (chromosome)	Rap10-F89: 5'-AGCGCGTCAAACGTGA<br>Rap10-R401: 5'-TCAGAAACGAGTGCAAGGT

BVH55_11275	RBAM_019730	Aspartate phosphatase, Rap11 (chromosome)	Rap11-F456: 5'-TGCTGAAGTGTACTATCACATG<br>Rap11-R718: 5'-ATGCCTTCTCCATATGTCCT
BVH55_14580	No homolog	Aspartate phosphatase, Rap12 (chromosome)	Rap12-F47: 5'-AGTGGTACAAGATGATTCGT<br>Rap12-R330: 5'-TTCGTACATTCCGCGAA
BVH55_18535	RBAM_033860	Aspartate phosphatase, Rap13 (chromosome)	Rap13-F91: 5'-CAAGCGGCTGATGCCA<br>Rap13-R424: 5'-GGAATTCCGCCCTCTCT
BVH55_16645	RBAM_030250	Protein LiaH	LiaH-F27: 5'-GTTCGTCGCATCTGTTAACG<br>LiaH-R301: 5'-CCGCTTTTCCCTCAAGGT
BVH55_16650	RBAM_030260	Protein LiaI	LiaI-F29: 5'-GCTTTCTGCTGATTGTGTTCG<br>LiaI-R311: 5'-GGTTCATACTGGCGGGAAG
BVH55_04520	RBAM_008370	RpiR family transcriptional regulator, GlvR	GlvR-F92: 5'-CGTGCTACCATCTCGGTATC<br>GlvR-R367: 5'-TCCCCGATCCGTAAACGA
BVH55_17385	RBAM_031680	Transcriptional regulator, SlrR	SlrR-F57: 5'-GCTAGCAGTTGAAGCAGGT<br>SlrR-R346: 5'-TTCTGTTGCGGTAGGGTG
BVH55_18750	RBAM_034290	Response regulator, Spo0F	Spo0F-F35: 5'-ACGGTATACGCATATTGCTCA<br>Spo0F-R341: 5'-ACCGCGTCTCTGATCTCA
BVH55_07570	RBAM_000460	AbrB family transcriptional regulator, AbrB	AbrB-F: 5'-TAGACGAACTCGGGCGT<br>AbrB-R: 5'-ACGCTTCCTCGATTTCCTC
BVH55_07575	RBAM_014230	PAS domain-containing sensor histidine kinase, KinC	KinC-F95: 5'-CGATTAACTGGCCCGTTGA<br>KinC-R431: 5'-TGCTGCTTGATTTGGGAGA
BVH55_06465	RBAM_012200	DeoR family transcriptional regulator, LacR	LacR-F45: 5'-CTCGCGGGGGTTTATTACG<br>LacR-R347: 5'-CGCTTATGCAGCAGGTGT
BVH55_16625	RBAM_030210	DNA-binding response regulator, LiaR	LiaR-F33: 5'-GATGGTCAGGATGGGTCTG<br>LiaR-R368: 5'-GGTTCACCCTTGGCAGC
BVH55_16630	RBAM_030220	Sensor histidine kinase, LiaS	LiaS-F107: 5'-ACGGGCTTGACCCGAT<br>LiaS-R394: 5'-AGAGCTTTTGCAGCGATGT
BVH55_05170	RBAM_009580	Histidine kinase FhlA, YhcY	YhcY-F110: 5'-TCAGCCTGACCGGACTG<br>YhcY-R470: 5'-GCTTCAAGCAGATGCAGTTC
BVH55_05175	RBAM_009590	DNA-binding response regulator, YhcZ	YhcZ-F33: 5'-TGTCCGCAAAGGACTGC<br>YhcZ-R353: 5'-TACACTTCCCGGAGCGT
BVH55_12035	RBAM_021250	PAS domain-containing sensor histidine	ResE-F134: 5'-ATTTAACGCAGCTTGCCGA

		kinase, ResE	ResE-R434: 5’-TACGGCACTCCGACGATC
BVH55_12040	RBAM_021260	DNA-binding response regulator, ResD	ResD-F46: 5’-GCCAGAATTCGACGCCTT<br>ResD-R340: 5’-CCACTTCTCTCGGGCTGA
BVH55_14460	RBAM_025960	DNA-binding response regulator, LytT	LytT-F147: 5’- TCTGCTGTTTTTGGACGTTGA<br>LytT-R432: 5’-ACCGGCTGATACAGCCA
BVH55_14465	RBAM_025970	Sensor histidine kinase, LytS	LytS-F274: 5’-ATCGGCGGACTCTTAGGC<br>LytS-R648: 5’-TCTCGCCCGTTCTTCCTT
BVH55_14550	RBAM_026140	PAS domain-containing sensor histidine kinase, PhoR	PhoR-F115: 5’-ACGGCGGATCAGCTGAA<br>PhoR-R456: 5’-TTCTCCGGTAACGCTTGAAC
BVH55_14555	RBAM_026150	DNA-binding response regulator, PhoP	PhoP-F65: 5’-TGGAACGGTCCGGCTAT<br>PhoP-R428: 5’-AGCTCGCCGATGAGGATC
BVH55_15185	RBAM_027320	Sensor histidine kinase, BceS	BceS-F170: 5’-ATCGGAAAGAGACCGCCTA<br>BceS-R490: 5’-GCTGCTGGTCCAGGAGA
BVH55_15190	RBAM_027330	DNA-binding response regulator, BceR	BceR-F96: 5’-GGATTTCAGCCGGGTCAT<br>BceR-R437: 5’-CGGCTGACCGTGTTGGT
BVH55_17925	RBAM_032640	DNA-binding response regulator, DegU	DegU-F92: 5’-TGGTAGCAGAAGGTGACGA<br>DegU-R400: 5’-TGTGGGTTACTTTCGGGTGA
BVH55_17930	RBAM_032650	Histidine kinase, DegS	DegS-F119: 5’-ATGAGCAGCTGGTTGAAGAA<br>DegS-R395: 5’-ATGATTTCCTGAAGCCCCAA
BVH55_15925	RBAM_028800	Degradation enzyme regulation protein DegQ	DegQ-F1: 5’-GTGGAAAACAAATTAGAAGAA<br>DegQ-R141: 5’-TTAAGAAATTTTCATTGCATATG
BVH55_15920		Isoprenyl transferase, ComQ	BVH-ComQ-F257: 5’-TTACGCTGAATACTGTAAC<br>BVH-ComQ-R519: 5’-TGATTGAACGATTGGGT
	RBAM_028790		FZB-ComQ-F251: 5’-ATGACATACAAGATAAGGATC<br>FZB-ComQ-R422: 5’-TCCATAGCTTGAACTGTA
BVH55_15915		Competence protein, ComX	BVH_ComX-F9: 5’-AATTGTAGGATACTTAACCAA<br>BVH_ComX-R162: 5’-TCAATATTTCCAATCACCGCC
	RBAM_028790		FZB_ComX-F23: 5’-TAGTTCGTAATCCCGAAA<br>FZB_ComX-R171: 5’-TGACGGTTTCCAATATATC
BVH55_15910	RBAM_028770	Histidine kinase, ComP	ComP-F1799: 5’-TAGAGGTGCAGGACAAG<br>ComP-R1980: 5’-CGTATTCAGACGGATATGAA

BVH55_15905	RBAM_028760	DNA-binding response regulator, ComA	ComA-F12: 5’-ACTAGTGATTGATGATCATC<br>ComA-R236: 5’-TTACAGAGAGGATTCTCTT
BVH55_02495	RBAM_004020	Phosphatase, PhrC	PhrC-F1: 5’-ATGAAATTGAAATCTAAATGGT<br>PhrC-R120: 5’-TCACGTCATTCCTCTTTCA
BVH55_18880	RBAM_034540	Phosphatase, PhrF	PhrF-F1: 5’-ATGAAATTGAAGTATAAATTAGCA<br>PhrF-R120: 5’-TTAAATCATTCCGCGCTGAGC
No locus tag	No homolog	Phosphatase, PhrF2	PhrF2-F1: 5’- ATGAAAACTAAACTATTTATCTGTG<br>PhrF2-R120: 5’- TTACGTTTCGGCTCTTT
BVH55_20090	RBAM_036900	Hypothetical protein, YxaL	YxaL-F169: 5’-GTTTCTTGGCGGGACGGT<br>YxaL-R446: 5’-AGCACCGGAGTCAGCGTA

Notes. Quantitative PCR primer sequences were designed based on the NCBI genome sequences of strain GH1-13 (CP019040.1) and FZB42 (CP000560.2). The PCR reaction with each primer set was performed using GoTaq qPCR Master mix (Promega, Madison, USA) incorporating a hot start at 95 °C for 2 min, followed by 30–35 cycles of melting at 94 °C for 15 s, annealing at 58 °C for 15 s, and elongation at 72 °C for 30 s.

Supplementary References

1. Darling, A. E., Mau, B. & Perna, N. T. progressiveMauve: multiple genome alignment with gene gain, loss and rearrangement. *PLoS One.* **5,** e11147 (2010).
2. Krzywinski, M. *et al.* Circos: an information aesthetic for comparative genomics. *Genome Res.* **19,** 1639-1645 (2009).
3. Johnson, M., Zaretskaya, I., Raytselis, Y., Merezhuk, Y., McGinnis, S. & Madden, T. L. NCBI BLAST: a better web interface. *Nucleic Acids Res.* **36,** W5-9 (2008).
4. Wu, C. H. *et al.* The Universal Protein Resource (UniProt): an expanding universe of protein information. *Nucleic Acids Res.* **34,** D187-D191 (2006).
5. Finn, R. D. et al. Pfam: the protein families database. *Nucleic Acids Res.* **42,** D222-D230 (2014).
6. McNeil, L. K. *et al.* The National Microbial Pathogen Database Resource (NMPDR): a genomics platform based on subsystem annotation. *Nucleic Acids Res.* **35,** D347-D353 (2007).
