# Supplementary material for: A native conjugative plasmid confers potential selective advantages to plant growth-promoting Bacillus velezensis strain GH1-13
Source: Commun Biol. 2021 May 14;4:582. doi: 10.1038/s42003-021-02107-z (PMC8121941; doi:10.1038/s42003-021-02107-z)
Supplement: Supplementary file 3 — Description of Supplementary Files [file 42003_2021_2107_MOESM3_ESM.pdf]

Description of Additional Supplementary Files

File Name: Supplementary Data 1

Description: BLAST analysis results for protein-coding DNA sequences in pBV71

File Name: Supplementary Data 2

Description: Annotations of protein-encoding genes in pBV71.

File Name: Supplementary Data 3

Description: Source data.

File Name: Supplementary Data 4

Description: Secretome analysis data.

File Name: Supplementary Data 5

Description: Transcriptome data analysis.

File Name: Supplementary Data 6

Description: Proteome analysis data.
