# Supplementary material for: A native conjugative plasmid confers potential selective advantages to plant growth-promoting Bacillus velezensis strain GH1-13
Source: Commun Biol. 2021 May 14;4:582. doi: 10.1038/s42003-021-02107-z (PMC8121941; doi:10.1038/s42003-021-02107-z)
Supplement: Supplementary file 10 — Reporting Summary [file 42003_2021_2107_MOESM10_ESM.pdf]

nature research

Corresponding author(s): Yong-Hak Kim

Last updated by author(s): Mar 30, 2021

Reporting Summary

Nature Research wishes to improve the reproducibility of the work that we publish. This form provides structure for consistency and transparency in reporting. For further information on Nature Research policies, see our Editorial Policies and the Editorial Policy Checklist.

Statistics

For all statistical analyses, confirm that the following items are present in the figure legend, table legend, main text, or Methods section.

n/a	Confirmed	
☐	☒	The exact sample size (n) for each experimental group/condition, given as a discrete number and unit of measurement
☐	☒	A statement on whether measurements were taken from distinct samples or whether the same sample was measured repeatedly
☐	☒	The statistical test(s) used AND whether they are one- or two-sided<br>*Only common tests should be described solely by name; describe more complex techniques in the Methods section.*
☐	☒	A description of all covariates tested
☐	☒	A description of any assumptions or corrections, such as tests of normality and adjustment for multiple comparisons
☐	☒	A full description of the statistical parameters including central tendency (e.g. means) or other basic estimates (e.g. regression coefficient) AND variation (e.g. standard deviation) or associated estimates of uncertainty (e.g. confidence intervals)
☐	☒	For null hypothesis testing, the test statistic (e.g. F, t, r) with confidence intervals, effect sizes, degrees of freedom and P value noted<br>*Give P values as exact values whenever suitable.*
☐	☒	For Bayesian analysis, information on the choice of priors and Markov chain Monte Carlo settings
☐	☒	For hierarchical and complex designs, identification of the appropriate level for tests and full reporting of outcomes
☐	☒	Estimates of effect sizes (e.g. Cohen's d, Pearson's r), indicating how they were calculated

Our web collection on statistics for biologists contains articles on many of the points above.

Software and code

Policy information about availability of computer code

Data collection	BioStudies accession number S-BSST608<br>NCBI BioProject PRJNA445855<br>PRIDE ProteomeXchange datasets PXD017305 and PXD008573
Data analysis	SigmaPlot-SPSS<br>String<br>Normalized Spectral Abundance Frequency (NSAF)<br>Trimmed Mean of M-values (TMM)

For manuscripts utilizing custom algorithms or software that are central to the research but not yet described in published literature, software must be made available to editors and reviewers. We strongly encourage code deposition in a community repository (e.g. GitHub). See the Nature Research guidelines for submitting code & software for further information.

Data

Policy information about availability of data

All manuscripts must include a data availability statement. This statement should provide the following information, where applicable:

- Accession codes, unique identifiers, or web links for publicly available datasets
- A list of figures that have associated raw data
- A description of any restrictions on data availability

Microscopic data are available under EMBL-EBI BioStudies accession number S-BSST608. Transcriptome data are available under NCBI BioProject PRJNA445855. Secretome and proteome data have been deposited into PRIDE and are available via ProteomeXchange under the dataset identifiers PXD017305 and PXD008573, respectively.

Field-specific reporting

Please select the one below that is the best fit for your research. If you are not sure, read the appropriate sections before making your selection.

- [x] Life sciences
- [ ] Behavioural & social sciences
- [ ] Ecological, evolutionary & environmental sciences

For a reference copy of the document with all sections, see nature.com/documents/nr-reporting-summary-flat.pdf

Life sciences study design

All studies must disclose on these points even when the disclosure is negative.

Sample size	Phenotype conversion and plasmid loss rate were determined using 300 randomly selected single colonies
Data exclusions	No data were excluded from the analyses
Replication	All cell culture experiments were repeated at least three times independently.
Randomization	Experiments that have been performed in three research teams supervised by three co-corresponding authors were double-blind randomized to receive strains, perform experiments, and evaluate the collected data.
Blinding	Researchers were blinded to receive strains, perform experiments, and evaluate the collected data.

Reporting for specific materials, systems and methods

We require information from authors about some types of materials, experimental systems and methods used in many studies. Here, indicate whether each material, system or method listed is relevant to your study. If you are not sure if a list item applies to your research, read the appropriate section before selecting a response.

Materials & experimental systems

n/a	Involved in the study	
[ ]	[x]	Antibodies
[x]	[ ]	Eukaryotic cell lines
[x]	[ ]	Palaeontology and archaeology
[x]	[ ]	Animals and other organisms
[x]	[ ]	Human research participants
[x]	[ ]	Clinical data
[x]	[ ]	Dual use research of concern

Methods

n/a	Involved in the study	
[x]	[ ]	ChIP-seq
[x]	[ ]	Flow cytometry
[x]	[ ]	MRI-based neuroimaging

Antibodies

Antibodies used	Western blot analysis was also performed using a rabbit polyclonal antibody for mature YxaL protein and a mouse monoclonal antibody raised against the C-terminal amino acid sequence (207-383 amino acid residues) of 3-phytase.
Validation	Kim et al. 2019. Plant growth-promoting activity of beta-propeller protein YxaL secreted from Bacillus velezensis strain GH1-13. PLoS One. 14, e0207968.<br>Ha et al. 2000. Crystal structures of a novel, thermostable phytase in partially and fully calcium-loaded states. Nat. Struct. Mol. Biol. 7, 147-153.<br>Details of the methods for western analysis using purified anti-YxaL and anti-PhyC IgG antibodies were described in the Methods section and full images of the western blots for YxaL and PhyC in the filtered (concentrated) culture fluids of wild-type and variant cells of Bacillus velezensis strain GH1-13 were shown in Supplementary Figure 12.
